# Analysis of clinical features of oxcarbazepine-induced Stevens-Johnson syndrome and toxic epidermal necrolysis

**DOI:** 10.3389/fmed.2023.1232969

**Published:** 2023-10-10

**Authors:** Qingzi Yan, Xiang Liu, Haibo Lei, Renzhu Liu, Yixiang Hu

**Affiliations:** Department of Clinical Pharmacy, Xiangtan Central Hospital, Xiangtan, China

**Keywords:** oxcarbazepine, drug adverse reaction, Stevens-Johnson syndrome, toxic epidermal necrolysis, severe cutaneous adverse reactions

## Abstract

**Background:**

Stevens-Johnson syndrome (SJS) is considered a hypersensitivity syndrome affecting the skin and mucous membranes. It has been reported that an anticonvulsant drug, oxcarbazepine, may cause Stevens-Johnson syndrome and toxic epidermal necrolysis (TEN). However, the clinical features of oxcarbazepine-induced Stevens-Johnson syndrome (SJS) and toxic epidermal necrolysis (TEN) remain ambiguous. This article aims to explore the clinical features of SJS/TEN.

**Methods:**

Systematic searches of several Chinese and English databases were conducted for case reports published on PubMed, EMBASE, Web of Science, MEDLINE, CNKI from January 1, 2007 to March 1, 2023.

**Results:**

A total of seventeen patients (10 males and 7 females) were included in this study, including nine adult patients and eight pediatric patients. The results showed that males seem to have a higher prevalence of SJS/TEN than females, and SJS/TEN usually occurs within 2 weeks after administration of oxcarbazepine (OXC). The main clinical manifestations among the included patients were rashes or maculopapules (17 cases, 100%), fever (11 cases, 64.7%), mucosal lesions (15 cases, 88.2%), conjunctivitis with/without ocular discharge (12 cases, 70.6%), and blisters (12 cases, 70.6%). After stopping OXC or switching to other drugs that treat primary disease as well as treatment with IVIG, glucocorticoid, anti-allergy, and fluid replacement, eight of the included patients recovered completely, and another eight of the included patients reported symptomatic improvement, while the prognosis of one of the included patients was not reported.

**Conclusion:**

Diverse clinical signs and symptoms of SJS/TEN might result in misinterpretation and delayed diagnosis. It should be identified and treated immediately to avoid significant consequences and potentially jeopardize patients’ lives.

## Introduction

As an anticonvulsant medication, oxcarbazepine (OXC) is structurally similar to carbamazepine (CBZ), which was initially utilized in patients with neuralgia and epilepsy. OXC is an inactive prodrug converted into a pharmacologically active intermediate — monohydroxycarbazepine (MHD). OXC and its metabolites can suppress repetitive neuronal discharge, stabilize the membrane of highly stimulated nerve cells, block voltage-sensitive sodium channels, and lessen the synaptic transmission of nerve impulses (1). Previous studies indicated that the most common adverse events of OXC were drowsiness, headache, dizziness, diplopia, nausea, vomiting, and fatigue, with an incidence of 10% (2). Over the past few years, it has been reported that OXC may cause Stevens-Johnson syndrome (SJS) and toxic epidermal necrolysis (TEN) (3).

SJS and TEN were described as serious mucocutaneous allergic reactions, mainly caused by certain medications such as antibiotics, NSAIDs (non-steroidal anti-inflammatory drugs), antiepileptics, or other medicines. SJS and TEN were categorized as belonging to the same spectrum of diseases and were classified according to the body surface area embroiled; skin injury surface area less than 10% is defined as SJS, more than 30% is considered as TEN and 10–30% is thought to be SJS-TEN overlap (4, 5). The primary clinical signs of SJS and TEN were maculopapules, which spread rapidly and merged to form blisters and epidermal necrosis. Despite the rare incidence of drug-induced SJS and TEN, the mortality rate is high, up to 7.5% in children and 20–25% in adults (6). In addition, the recovery period of SJS/TEN may also leave sequelae, including cutaneous problems (84.3%), ocular problems (59.5%), and oral mucosal problems (50.8%) (7). Because of the severity of SJS/TEN, early identification, diagnosis, and therapy of SJS/TEN are incredibly significant in reducing mortality. However, the timely identification and effective management of this condition are challenges for healthcare professionals. The clinical features reported in different case reports are inconsistent and variable and the diagnosis and treatment of SJS/TEN vary greatly among different specialists, and the data related to the treatment outcome are limited. There is still a lack of adequate evidence-based evaluation for OXC-induced SJS/TEN. This review aimed to investigate the clinical features of OXC-induced SJS/TEN by collecting relevant cases to provide a reference for accurate diagnosis, appropriate treatment, and prognosis of SJS/TEN.

## Methods

### Search strategy

The oxcarbazepine induced SJS/TEN case reports were collected by searching Chinese and English databases, including CNKI, Pubmed, Embase, and Web of Science, from 2007 to March 1, 2023. The following search terms were used to establish the search strategy, including ‘Oxcarbazepine’, ‘10,11-Dihydro-10-oxo-5H-dibenz(b,f)azepine-5-carboxamide’, ‘Trileptal’, ‘Timox’, ‘GP 47680’, ‘OXC’, ‘MHD’, ‘Monohydroxycarbazepine’ and ‘Stevens-Johnson Syndrome’, ‘Stevens Johnson Syndrome’, ‘Toxic Epidermal Necrolysis’, ‘Drug-Induced Stevens Johnson Syndrome’, ‘SJS’, ‘Epidermal Necrolysis, Toxic’, ‘Toxic Epidermal Necrolyses’, ‘TEN’.

### Inclusion and exclusion criteria

Inclusion criteria: (1) studies published by case reports of OXC-induced SJS and TEN. (2) the full article was written in Chinese or English.

Exclusion criteria: studies published by review, mechanistic studies, only abstract, no full-text researches, animal studies, and duplicate cases.

### Data extraction

The data extraction was conducted independently by two reviewers using self-designed tables. Data were extracted on the first author, publication year, age, disease history, primary disease treatment drugs, the onset time after administration, clinical manifestations, laboratory examinations, body examination, treatment, and prognosis.

## Results

### Basic information

**Figure 1:**
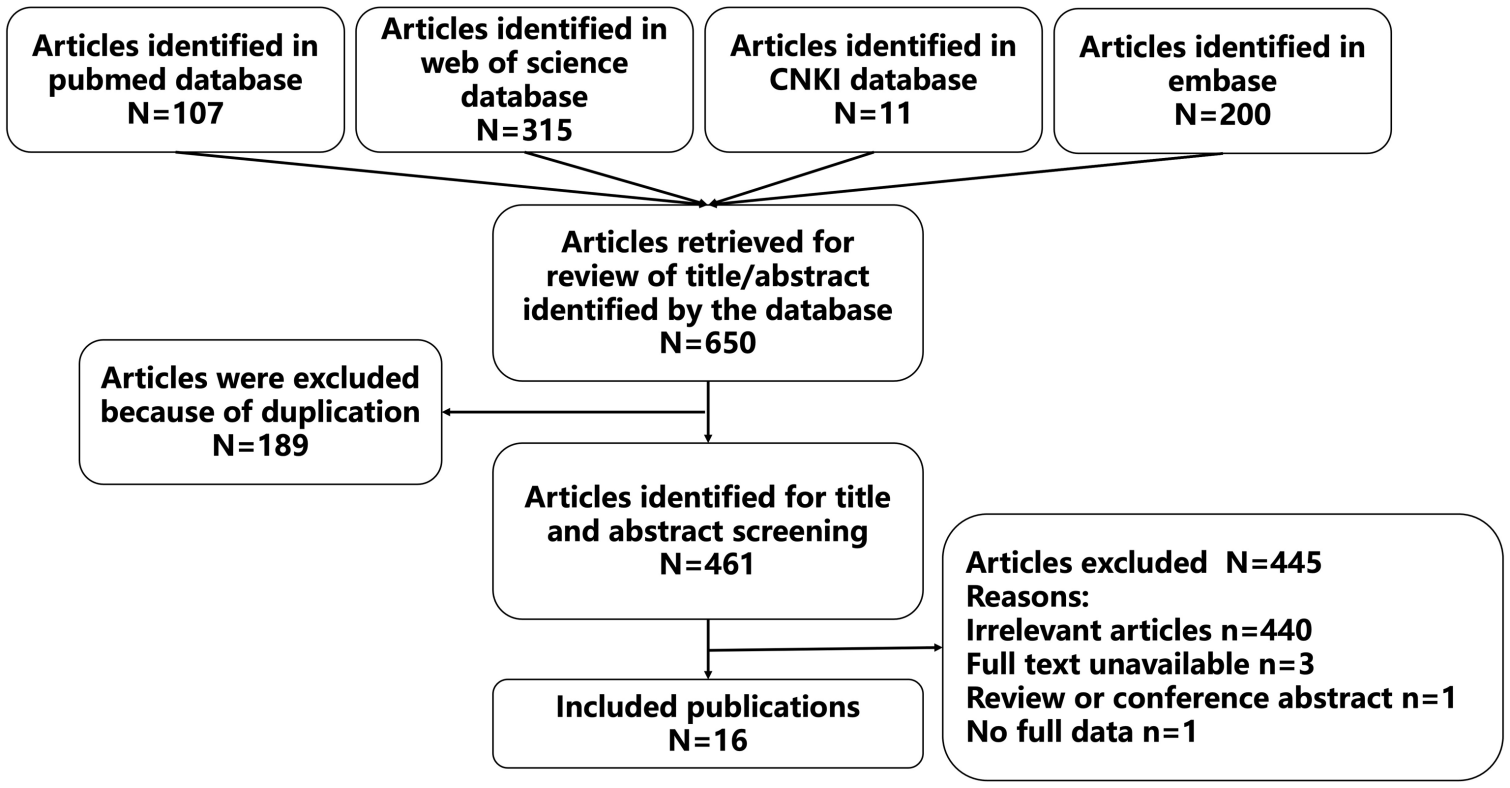
Article selection methodology.

Sixteen studies were included according to the inclusion and exclusion criteria. The details of the search results were shown in Figure 1, and information for these studies were presented in Table 1. Of the 16 included studies, eleven of them were reported in English, and five of them were reported in Chinese. A total of 17 patients were reported in the 16 included studies (including 10 males and 7 females), who were from China (8 cases, 47.1%), India (5 cases, 29.4%), USA (1 case, 5.9%), Mexico (1 case, 5.9%), Peru (1 case, 5.9%), and Turkey (1 case, 5.9%). In terms of age, nine of the included patients were adults (52.9%) and eight of them were pediatric patients (47.1%). In addition, 15 of the cases were treated with OXC because of epilepsy (88.2%), one cases was treated with OXC because of trigeminal neuralgia (5.9%), and one case was treated with OXC because of oppositional defiant disorder and a bipolar disorder variant (5.9%). The primary diseases of 11 patients were treated with other drugs and received or added OXC treatment due to poor therapeutic effect and other reasons.

**Table 1:** Basic information of the 17 patients included.

Reference	ID	Country	Age (years)	Sex	Disease history	History of drug use or combination therapy	OXC dose	Time of onset (days)
(8)	1	Turkey	6	Male	Epilepsy	na	12 mg/kg/day→18 mg/kg/day	10
(9)	2	China	10	Male	Epilepsy	VPA	150 mg bid	9
(10)	3	Taiwan, China	53	Male	Hypertension and cerebral vascular accidents, episodes	Enalapril maleate, amlodipine besylate and aspirin, PB, VPA	na	11
(11)	4	India	60	Male	Hypertensive, seizures	Losartan, PHT	300 mg bid	14
(12)	5	USA	20	Male	Oppositional defiant disorder and a bipolar disorder variant	na	300 mg bid→600 mg bid	9
(13)	6	Taiwan, China	9	Male	Seizure	PHT	300 mg qd → 300 mg bid	14
(14)	7	Peru	81	Female	Trigeminal neuralgia	CBZ	300 mg tid	11
(15)	8	Mexico	10	Female	Epilepsy	OXC	10 mg/kg/day	22
(16)	9	India	21	Male	Epilepsy	na	300 mg bid→600 mg, bid	14
(17)	10	India	18	Female	Epilepsy	Clonazepam	200 mg qd	7
(17)	11	India	58	Male	Epilepsy	PHT	na	4
(18)	12	India	38	Female	Seizure	na	600 mg/day→900 mg/day	12
(19)	13	China	3	Female	Seizures	LEV, topiramate	25 mg/kg/d	14
(20)	14	China	6	Female	Epilepsy	LEV	0.5 tablets/d	17
(21)	15	China	29	Male	Epilepsy	na	4 tablets/d	10
(22)	16	China	3.5	Male	Seizures	VPA，ceftriaxone, azithromycin	20 mg/kg/d → 25 mg/kg/d	6
(23)	17	China	6	Female	Epilepsy	na	100 mg bid	14

OXC, Oxcarbazepine; VPA, Valproic acid; PHT, Phenytoin; CBZ, carbamazepine; LEV, Levetiracetam; LTG, Lamotrigine; PB, Phenobarbital; na, not available.

### Administration of oxcarbazepine

Two patients reported that the drug dose was calculated in units of tablets, and no accurate dose was reported (2 cases, 11.8%). The dose of 4 pediatric patients was calculated by body weight; the initial dose of OXC ranged from 10 mg/kg/day- 20 mg/kg/day, then the dose was increased to 18 mg/kg/day-25 mg/kg/day within 1 week. For the other patients, the daily dose of OXC was used at a standard dose(100-900 mg/day), and no overdose were used. The precision dose of OXC for different patients were shown in Table 1. Most of the patients (15 cases, 88.2%) developed SJS/TEN within 2 weeks after OXC exposure, of which three patients (3 cases, 17.6%) developed SJS/TEN within 1 week. SJS/TEN occurred in the other three patients (2cases, 11.8%) who treated with OXC for over 2 weeks.

### Diagnosis and adverse reaction causality evaluation

All the included patients were diagnosed as SJS/TEN according to clinical symptoms and laboratory tests after consultation in the department of dermatology. Of the included patients, fifteen of them (15 cases, 88.2%) were diagnosed as SJS, one patient (1 cases, 5.9%) were diagnosed with SJS/TEN overlap, and one patient (1 case, 5.9%) was diagnosed as TEN. Moreover, SJS occurred in seven patients (7 cases, 41.2%) after OXC monotherapy because of epilepsy and one patient (1 case, 5.9%) developed SJS after taking OXC due to mental illness. Six patients (6 cases, 35.3%) experienced SJS after converting to OXC from other antiepileptic medications due to poor seizure control of the initial drug and two patients (2 cases, 11.8%) developed SJS after adding OXC. One patient developed SJS after turning from CBZ to OXC due to trigeminal neuralgia.

### Clinical symptoms

The majority of patients were prescribed OXC for seizure control and SJS/TEN was observed within 4–22 days (median time was 11 days) after exposure to OXC. When SJS/TEN occurs, the main clinical symptoms were as follows: rashes or maculopapule (17 cases, 100%), fever (11 cases, 64.7%), mucosal lesions including oral cavity, genitalia, and nose (15 cases, 88.2%), conjunctivitis with/without ocular discharge (12 cases, 70.6%), blisters (12 cases, 70.6%). Moreover, some patients also have other symptoms such as myalgia and pain on swallowing，flat atypical target lesions with central dusky discoloration, Headache, myalgia, fatigue and Lymphadenopathy, etc. The clinical symptoms of the 17 patients were summarized in Table 2.

**Table 2:** Clinical symptoms of the 17 included patients.

ID	Diagnosis	Clinical symptoms					
		Fever	Rashes/maculopapule	Mucosal lesions (oral cavity, genitalia, nose)	Conjunctivitis	Bullousand/vesicles/blisters	Others
1	SJS	na	√	√	na	√	Myalgia and pain on swallowing
2	SJS	√	√	√	√	na	Atypical target lesions with central dusky discolouration
3	SJS	√	√	√	√	na	Flat atypical target lesions with central dusky discoloration
4	TEN	√	√	√	√	√	na
5	SJS	√	√	√	√	√	Headache, myalgia, fatigue, sore mouth and throat
6	SJS	√	√	√	√	√	na
7	SJS/TEN	na	√	√	na	√	Skin detachment
8	SJS	na	√	√	√	√	na
9	SJS	√	√	√	√	√	na
10	SJS	na	√	na	na	na	Lymphadenopathy
11	SJS	na	√	√	na	na	na
12	SJS	√	√	√	√	√	Sore throat, fatigue, blisters
13	SJS	√	√	√	√	√	Lymphadenopathy
14	SJS	√	√	√	na	√	
15	SJS	na	√	na	√	√	Pulmonary infection, myocardial enzyme index increased significantly, oliguria
16	SJS	√	√	√	√	na	na
17	SJS	√	√	√	√	√	Urinary pain and sore throat

### Laboratory tests

Two patients (2 cases, 11.8%) were reported with no significant changes in whole blood cell count and biochemical markers. Three patients (3 cases, 17.6%) were genotyped for HLA-B*1502, and two patients (2 case, 11.8%) were genotyped for HLA-B*1518/B*4001. Infection indicators such as white blood cells, CRP, and granulocytes were increased in 8 patients (8 cases, 47.1%). Seven patients (7 cases, 41.2%) were performed skin biopsy to further confirm the SJS/TEN, the primary histological characteristics were focal full thickness epidermal necrosis, basal vacuolar changes, and perivascular lymphocytic infiltrates in the papillary dermis, positivity with CD3 and CD8 antibodies. Partial of patients who did not perform skin biopsy were diagnosed by dermatologists after consultation with clinical symptoms and other laboratory tests.

### Treatment and prognosis

The treatment and prognosis of the 17 patients were summarized in Table 3. After developing SJS/TEN, OXC was discontinued or switched to other antiepileptic drugs, including LEV, LTG, and VPA in eleven patients (11 cases, 64.7%). It was not reported whether the other 6 patients discontinued OXC. The other most common therapies for SJS/TEN were steroids (13 cases, 76.5%), antihistamine (10 cases, 55.8%), and IVIG (6 cases, 35.3%). Moreover, total parenteral nutrition, electrolyte, antibiotics, oral care, and other supportive therapy were used to treat the SJS/TEN. Finally, eight patients (8 cases, 47.1%) recovered completely, the symptoms of eight patients (8 cases, 47.1%) were improved, and the prognosis of one patient was not reported.

**Table 3:** Laboratory examination, treatment, and prognosis of 17 patients included.

ID	Laboratory examination	Treatment	Prognosis
1	Skin biopsy: focal full-thickness epidermal necrosis, basal vacuolar changes and perivascular lymphocytic infiltrates in the papillary dermis, positivity with CD3 and CD8 antibodies	OXC withdrawal, topical steroid	Symptoms improvement and discharge after 7 days
2	Genotype: HLA-B*1502, other laboratory results were normal	Steroid, azithromycin, and cream urea was applied on lips, tobramycin-dexamethasone eyedrops	Completely recovered after 15 days
3	Genotype: HLA-B*1502, skin biopsy: necrosis of the whole epidermis with sparse dermal infiltrates of inflammatory cellsp	na	na
4	Leukocytosis, elevated CRP, skin biopsy: full thickness necrosis of the epidermis	OXC withdrawal and switch to LEV, steroid, anti-H1, and H2 receptor blockers	Recovery after 2 weeks
5	Elevated CRP, leukocytopenia, biopsy and histologic examination	OXC withdrawal, IVIG	Symptoms complete resolution in 1 week.
6	Leukocytosis, elevated CRP, skin biopsy: liquefactive degeneration in the lower half of the epidermis with some dyskeratotic keratinocytes, CD8+ lymphohistiocytic infiltration around the blood vessels and scanty eosinophils, genotype: HLA-B*1518/B*4001	Steroid, antihistamine	Symptoms improved and discharged after 12 days.
7	na	Steroids, antibiotic, antihistaminic	Restore favorably
8	na	OXC withdrawal, IVIG, clindamycin,	Marked improvement after 8 days treatment
9	Leucocytosis elevated, elevated CRP, skin biopsy: there was marked liquefactive degeneration with some dyskeratotic keratinocytes. The dermis showed lymphohistiocytic infiltration around blood vessels and scanty eosinophils	Intravenous steroid, antihistamine.	Symptoms improved discharged after 14 days
10	na	OXC withdrawal, intravenous steroid, pheniramine, ranitidine, paracetamol, clotrimazole local application	Recovered in 12 days
11	na	OXC withdrawal, intravenous steroid, levocetirizine, calamine, mupirocin 2%, gatifloxacineye	Recovered after 13 days
12	Leukocytosis, elevated CRP, genotyping: HLA-B*1518/B*4001, skin biopsy: there was marked liquefactive degeneration in the lower half of the epidermis with some dyskeratotic keratinocytes. The dermis showed predominant CD8+ lymphohistiocytic infiltration around the blood vessels and scanty eosinophils.	Corticosteroids, ntihistamine	Symptoms improved after 10 days
13	Leukocytosis, neutrophils increased, CRP increased, ESR accelerated.	OXC withdrawal, steroid, IVIG, antihistaminic, liver, gastric mucosal protection, oral care, nutritional support	Symptoms improved after 10 days
14	na	OXC withdrawal, steroid, cefazolin, loratadine, potassium chloride, calcium gluconate oral liquid, Triamcinolone acetonide econazole cream	Completely recovered after 3 weeks
15	Elevated myocardial enzymes, Genotype: HLAB*1502	OXC withdrawal and switch to LTG, anti-infection, desensitization, myocardial nutrition, diuresis, skin care and supportive treatment	Completely recovered after 1 month.
16	Leukocytosis, neutrophils increased, CRP increased, ESR accelerated	OXC withdrawal and switch to VPA. intravenous steroid, IVIG, 10% calcium gluconate, V_C_, loratadine syrup	Completely recovered after 1 month
17	Elevated CRP, increased procalcitonin	OXC withdrawal and switch to LEV, ceftriaxone, intravenous steroid, IVIG, antihistamine, PPI	Symptoms improved after 17 days

V_C_, vitamin C; IVIG, intravenous immunoglobulin; PPI, ProtonPump Inhibitors; CRP, C-reactive protein; ESR, erythrocyte sedimentation rate.

## Discussion

### Key findings

It is generally believed that OXC induced cutaneous adverse reactions less than CBZ (24). According to the previous report, the estimated relative risk of CBZ-SJS was 30 to 40 fold than that of OXC-SJS among Han Chinese in Taiwan, the incidence of CBZ-SJS was 2.6–3.4 cases per thousand person-years and OXC-SJS is only 0.08 cases per thousand person-years (10). VPA, LTG, CBZ, and other antiepileptic drugs can also cause SJS/TEN, with the highest absolute risk among new users of LTG and PHT (both approximately 45 cases/100,000 new users), followed by CBZ (20 cases/100,000 new users) (25). In addition, SJS/TEN may induced by some macrolide antibiotics such as azithromycin, clarithromycin, erythromycin, roxithromycin or telithromycin (26). However, there is a lack of research on whether there are differences in the clinical characteristics of SJS/TEN caused by different types of materials. More research is needed to further explore the clinical characteristics of SJS/TEN caused by different types of substance, so that to stop suspicious materials in time when patients take multiple drugs.

As SJS/TEN occurs, the risk of secondary infection, multiple organ failure, liver and kidney damage, aplastic anemia, and about half of the patients may have long-term skin and eye sequelae, and even mortality is exceptionally high; with early treatment, the survival rate for SJS/TEN patients may exceed 90% (27). But, the diagnosis of SJS/TEN is based on the clinical manifestations of acute episodes, including rapidly expanding target-like erythema, epidermal necrosis and exfoliation, and erythema, erosion, and crusting on two or more mucosal surfaces，remains difficult after excluding other interference factors such as SSSS (staphylococcal scalded skin syndrome), TSS (toxic shock syndrome), MCLS (mucocutaneous lymph node syndrome)，autoimmune disease. In addition, attention should be paid to the differential diagnosis between SJS/TEN, DRESS (drug reaction with eosinophilia and systemic symptom) and AGEP (acute generalized exanthematous pustulosis). DRESS and AGEP can be caused by antimicrobials, antipyretic and analgesic agents, antihypertensive medications, chemotherapeutic drugs, biological preparation antiepileptic drug and other drugs (28–30). Clinically, AGEP is characterized by pinpoint-sized non-follicular aseptic pustular plaques based on erythema, as well as systemic symptoms such as leukocytosis and elevated neutrophils, with no or minimal damage to the mucous membranes (31). DRESS is characterized as an adverse drug reaction based on erythema, fever, lymph node enlargement, and eosinophilia with variable system and organ involvement (32). In OXC induced-SJS/TEN patients, who treated with recommended dose of OXC, indicating that SJS/TEN may not be related to the dose of OXC. The primary clinical symptoms are dermatological manifestations such as reddish pruritic rashes, erythematous maculopapular eruptions, mucosal lesions on the lip, nose, and exulcerations in the genital region and then fusion to form bullae or blisters, as well as the non-dermatological manifestation such as high fever, myalgia, conjunctivitis, CD8^+^ antibody positive, white blood cells, CRP, neutrophils and other inflammatory indicators increased leading to pain, unable to eat, loss of a large number of body fluids and electrolytes (26, 33). When mucosal lesions occur in severe cases of AGEP or SJS/TEN-like exfoliative dermatitis occurs in patients with severe DRESS, it may be difficult to distinguish SJS/TEN among them. Generally, AGEP typically undergoes eruption within 24–48 h, compared with a longer incubation period within 2–6 weeks for DRESS (28), whereas the results in this study showed that SJS/TEN generally occurs within 4–22 days after OXC exposure, the median time was 11 days, which was shorter than the instructions’ recorded time (a median of 19 days). The median time reported in our study was also shorter than the median time in Mockenhaupt’s study on CBZ (15 days), PHT (24 days), VPA (more than 30 weeks), phenobarbital (17 days) (34). In terms of cutaneous features, SJS/TEN presented as bullae and mucosal (including oral cavity, eye, and genital) lesions, AGEP is characterized by sterile pustules on erythematous base with minimal mucous membrane involvement, the skin features of DRESS are morbilliform diffuse, pruritic, macular exanthema. In histopathology, the OXC-SJS/TEN patients in this study were mainly manifested as focal full thickness epidermal necrosis, basal vacuolar changes, and perivascular lymphocytic infiltrates in the papillary dermis, while DRESS exhibits perivascular lymphocytic infiltration, and AGEP shows intracorneal, subcorneal, or intraepidermal pustules according to previous study (28).

### Pathogenesis of OXC-SJS/TEN

Medications induce more than 80% of SJS/TEN; at least 200 drugs including OXC may cause SJS/TEN (35), AEDs are among the most frequently reported triggers of SJS/TEN. However, the SJS/TEN pathogenesis is still not completely clarified. It is acknowledged that the apoptosis of keratinocytes is one of the mechanisms, which involved in the Fas/FasL interaction, cytotoxic T cells, TNF-α, and nitric oxide synthase (36). Studies have shown that pathogenesis of drug-induced SJS/TEN is abnormal drug metabolism in some patients, who have different detoxification abilities to active metabolites, or this active metabolite may directly produce toxicity or may form antigens with host tissues, thereby inducing T cell-mediated cytotoxicity of intracellular drug antigens (37). OXC is a structural derivative of carbamazepine, it is rapidly and almost completely converted into pharmacologically active metabolites(10-monohydroxy derivatives, MHD) under the catalysis of liver cell enzymes after taking OXC, and further metabolized by the glucuronate pathway, about 4% of the OXC was oxidized to non-pharmacological activity metabolites─10,11-dihydroxy derivative (DHD) (38). Moreover, CBZ, LTG, PB, and PHT are all metabolized to arene oxide metabolites, these highly reactive intermediate metabolites binding with cellular macromolecules to produce toxic antigens, it is considered to be a type 4 hypersensitivity reaction, which have been hypothesized to cause skin adverse reactions (39). Additionally, there may be cross hypersensitivity between these drugs that the metabolites are arene oxide metabolites. For previous SJS/TEN patients, some AEDs (LEV, topiramate, clonazepam, etc.) may be safer options for antiepileptic therapy (25). Consistent with the results of our study, SJS/TEN did not trigger again when OXC was switched to another antiepileptic drug in some patients. Hence, it may be considered to switch to medications whose metabolites are not arene oxide metabolites, such as LEV, topiramate, and clonazepam, but more scientific evidence is needed to support this. It has also been reported that SJS/TEN is associated with the cell-mediated inflammatory response and immune system. Macrophages, CD8^+^ T cells, CD25^+^ T cells, and CD4^+^ T cells in the epidermis and dermis release inflammatory factors such as interferon-γ and TNF-α to promote an immune response to epidermal and keratin damage (40). Part of the patients included in this study did not perform histopathological examination, and the patients who examined histopathology were mainly manifested as focal full thickness epidermal necrosis, basal vacuolar changes, and perivascular lymphocytic infiltrates in the papillary dermis.

Moreover, genotype is closely related to the occurrence of SJS/TEN, especially when the patient’s genotype is HLA-B*1502, who had the higher incidence of SJS/TEN. In Asian populations, the risk of SJS/TEN seems to be higher in patients with HLA-B*1502 (41), (OR = 27.90; 95% CI: 7.84–99.23) in Chinese. The positive and negative predictive values of HLA-B*1502 for OXC-SJS/TEN were 0.73 and 99.97%, respectively. The incidence and mortality of OXC-SJS/TEN was lower than CBZ-STS/TEN in new users (*p* = 0.003; relative risk 0.212; 95%CI 0.077–0.584) (42). Moreover, it is worth noting that HLA-B*1502 is associated with OXC-SJS/TEN but not OXC-DRESS, and HLA-A*3101 has been previously reported to be associated with CBZ-DRESS; however, there was no significant association with OXC-DRESS in Asians (42, 43). Therefore, it is necessary to detect the genotype of patients when taking drugs that may cause SJS/TEN such as OXC, LTG, VPA, etc. If the genetic information were known *a priori*, physicians can formulate a personalized treatment plan based on this to prevent SJS/TEN. The primary treatment for SJS/TEN is discontinuation of OXC, and other treatments include pulsed corticosteroids and intravenous IVIG in some severe patients. Secondly, supportive treatment is the focus of treatment for SJS/TEN patients, including fluid and electrolyte supplementation, infection control and trauma care. Most patients can completely recover after reasonable and timely treatment. For patients who still need antiepileptic, the alternative is to switch to other relative drugs such as pregabalin, LEV, and VPA, etc.

### Limitations

This study has some limitations such as cannot be used to determine the incidence of events due to the fact that it was based on available cases in the literature. Secondly, we excluded studies that were not written by English and Chinese. The quality of most of these included studies was low or moderate, mainly due to the lack of a large amount of important information in these case reports such as SJS/TEN was diagnosed with the assistance of dermatologists, some patients did not perform skin biopsy. Thirdly, these cases from diverse clinical setting, and missing some important information. There are differences in diagnostic tools, medical standards, and reporting quality result in the data in this article may still be biased. We should call on researchers to provide as much detailed information as possible improve the quality of case reports when reporting cases. Nevertheless, this study provides some preliminary insights into the clinical features of SJS/TEN patients caused by OXC, which helps in the improved recognition and management of OXC-induced adverse reactions.

## Conclusion

To sum up, SJS/TEN is a rare complication of OXC. Patients treated with OXC should always pay attention to their symptoms, such as unexplained rash or maculopapule, fever, oral ulcer, and other symptoms. If any of the symptoms mentioned above appear when OXC is being administered, the medicine should be stopped immediately and followed up regularly throughout the medication period. Clinicians should promptly identify SJS/TEN when skin or fever-related adverse reactions occur so that timely intervention and early treatment minimize the deleterious influences of OXC induced-SJS/TEN.

## Data availability statement

The original contributions presented in the study are included in the article/supplementary material, further inquiries can be directed to the corresponding author.

## Author contributions

QY designed the study, wrote the manuscript, and prepared the original draft. XL provided the clinical support and reviewed the final version of the manuscript. RL and HL collected the data. YH analyzed the data. All authors have agreed to the submission of the final manuscript.
